# The Epidemiology of Bovine Viral Diarrhea Virus in Low- and Middle-Income Countries: A Systematic Review and Meta-Analysis

**DOI:** 10.3389/fvets.2022.947515

**Published:** 2022-08-03

**Authors:** Bibiana Zirra-Shallangwa, Lina González Gordon, Luis E. Hernandez-Castro, Elizabeth A. J. Cook, Barend M. de Clare Bronsvoort, Robert F. Kelly

**Affiliations:** ^1^The Royal (Dick) School of Veterinary Studies, The Roslin Institute, University of Edinburgh, Easter Bush, Midlothian, United Kingdom; ^2^Centre for Tropical Livestock Genetics and Health, International Livestock Research Institute, Nairobi, Kenya

**Keywords:** bovine viral diarrhea (BVD), BVDV, risk factors, health impact, economic impact, LMICs

## Abstract

**Introduction:**

Bovine viral diarrhea virus (BVDV) causes reproductive inefficiencies and negatively impacts the economy of low- and middle-income countries (LMICs). It is characterized by a combination of syndromes that result in poor production performance and calf morbidity and mortality. BVDV control is possible by introduction of biosecurity measures, test-and-cull, and vaccination programs as accomplished in high-income countries. Knowledge of BVDV epidemiology is limited in many LMICs, which hinders implementation of effective control programs. We carried out a systematic review and meta-analysis to estimate the burden of BVDV, identify risk factors related to its occurrence, and health and economic impacts on production systems.

**Materials and Methods:**

Relevant BVD articles were collated from library databases; 690 abstracts and full texts were found in an initial search followed by filtering of 59 manuscripts. We accounted for quality and risk of bias in the meta-analysis. Prevalence, exposure, and current infection at regional, production, and farming system levels were estimated using logistic random-effects meta-regression models. Finally, we calculated the proportion of studies that addressed risk factors and health and economic impacts across different production systems to inform future preventative strategies in LMICs.

**Results:**

Seroprevalence was high and varied between regions. Mean weighted prevalence was 39.5% (95% CI 25–56.1), 45.2% (95% CI 35.9–54.8), 49.9% (95% CI 25.5–74.3), and 21.6% (95% CI 0.5–56) for sub-Saharan Africa, South America, Middle East, and Asia, respectively. Seroprevalence varied across farming systems, with smallholder farming showing the highest values. Herdsize was the most frequently reported risk factor, and the percentage of articles that reported herdsize as a risk factor were 20.6%, 33.3%, and 38.4% for dairy, beef and mixed systems respectively. Abortion (13.7% of articles) was the main reported health impact in dairy systems. Some articles reported milk drop (4.6% of articles), but no article investigated the economic cost of BVDV in farming systems.

**Conclusion:**

Animal-level seroprevalence varied across all regions. Most of the studies focused on BVDV seroprevalence. There were some articles that investigated risk factors and health impacts, and there were even less that investigated economic impacts. Future studies should focus on identifying risk factors and quantifying health and economic impacts across systems. Understanding these aspects is crucial to develop management strategies to apply across diverse production systems in LMICs.

## Introduction

Bovine viral diarrhea virus (BVDV) causes a pathogenic infection in livestock, mainly cattle and wild ruminants, with a global distribution (1, 2) resulting in abortion, calf mortality, and poor reproductive performance. Livestock production and keeping play a significant role in poverty and hunger alleviation in many countries around the world (3). Livestock in LMICs provide an important food source, drought power, and manure source, as well as regular monetary income. Current meat and milk demand exceeds livestock production for growing populations in LMICs (4–6). BVDV impacts negatively on animal production and population livelihood (6). Unlike other major infectious diseases, such as foot-and-mouth disease or brucellosis, there have been few efforts to understand BVD in LMIC settings and develop pathways for control or eradication (7).

BVDV is a member of the *Flaviviridae* family in the genus *Pestiviruses* and has 2 main types reported, BVDV-1 and BVDV-2. Recently, there has been a BVDV-3 or HoBi-like virus found in several regions (1, 8); however, this has not yet been reported in sub-Saharan African (SSA) countries (1, 9). BVD-1 and BVD-2 have two subtypes referred to as the cytopathic or cell-killing form and the non-cytopathic form which infects cells but does not cause cell death (10). Both of the subtypes however, still cause disease in animals leading to reproductive and economic losses. Specifically, losses, such as abortion, poor reproductive performance, low milk yield, and high calf morbidity and mortality have been quantified in high-income countries (11) but poorly monitored in LMICs. BVD is widespread, and prevalence varies across regions, i.e., in sub-Saharan Africa, antibody prevalence is between 51 and 77% (12–14), and antigen prevalence is up to 19%, while in Europe, antibody prevalence is about 46% and antigen prevalence is about 0.2% (15). The variations in prevalence are influenced by a variety of factors, including farming systems and options for control in different areas.

Transmission of the disease is mainly driven by presence of persistently infected (PI) animals in a herd or contact with them. PI animals are infected *in utero* during gestation, usually between days 18 and 125 (16), such that they do not develop an immune response to the virus but become persistently viremic and then shed the virus after birth (1, 10). Animals can also be transiently infected (TI) for a short period (usually between 2 and 3 weeks) and can shed the virus for a short time before they mount an immune response and then clear the infection (10, 17). Presence of infection is usually maintained in a herd because of ongoing production of PI animals and then shedding large amounts of the virus infecting naïve animals. Clinical outcomes of BVD depend on the host, stage of pregnancy, and strain of the infecting BVDV. Infection usually results in signs, such as transient viremia associated with leukopenia, thrombocytopenia, cell death in the thymus, pyrexia, and diarrhea leading to immunosuppression, which allows for co-infections such as respiratory pathogens plus fetal loss, including abortion, and fetal abnormalities in pregnant animals (8, 17, 18). Lack of pathognomonic clinical signs and the more chronic nature of the disease make it harder to diagnose in farms. Serum antibody diagnostic tests such as enzyme-linked immunosorbent assays (ELISAs) can be conducted to detect animals exposed after birth, but PI animals exposed *in utero* (between days 18 and 125 of gestation) do not produce antibodies and can only be detected by identifying the virus using either antigen ELISAs or molecular tools such as polymerase chain reactions (PCRs) (84, 85). Many high-income countries such as the United Kingdom have eradication programs using combinations of ELISAs and PCR to detect and remove PI animals from infected herds, vaccination (to prevent infection during pregnancy), test-and-cull, and implementation of biosecurity measures (9–11, 19). The diagnostic tests available for testing BVD-specific antibody and antigen are generally very reliable (20) and reported to have excellent sensitivity and specificity (10, 21–23). The accuracy of diagnostic tests helps give a good understanding of the epidemiology of BVD. Diagnostic tests are also essential components of an eradication program, and their accuracy has made BVD control programs economically achievable (19).

The potential impact of BVDV infection is well-recognized in high-income countries where its epidemiology is well-understood and its economic impacts are well-described and quantified. For example, in North American systems, it has been estimated that infection with BVDV can cost up to US$88/animal in beef herds due to calf mortality which leads to considerable financial losses for individual farmers (2, 24). Acute infections have been shown to cause up to a 23% milk drop in the 2 weeks following infection in United Kingdom dairy herds (25). The global distribution of BVDV (26) and its reported associated reproductive losses highlight why the virus has now been listed as a class B disease by the Office International des Epizooties (OIE) (27), and a reportable disease in cattle only and not in multiple species (1). There is, however, a gap in our understanding of the epidemiology of BVDV and its health and economic impacts on LMICs that need to be addressed urgently to reduce inefficiencies in livestock production that lead to antibiotic misuse and contribute to climate change, and to improve animal welfare. There are a few studies that have been conducted to determine the seroprevalence of BVDV antibodies, and it was our goal to bring together these studies in a systematic review in an attempt to understand the epidemiology and quantify the importance of risk factors and economic impacts of BVDV on cattle-rearing communities in LMICs.

Systematic reviews are an important tools for collating data from multiple published studies to improve parameter estimates such as prevalence and to identify research gaps (28, 29) and may be an important resource to policymakers when considering the design of disease control programs. Meta-analysis helps to give a precise estimate of the overall or combined effects of studies (30). This systematic review and meta-analysis of BVDV epidemiology in LMICs aims to (1) summarize the available data on the prevalence of BVDV, (2) describe the common risk factors and BVDV's health and economic impacts on LMICs, and (3) identify and collate gaps in knowledge for future research.

## Materials and Methods

This systematic review followed the guidelines and checklist of the PRISMA Group (PRISMA Transparent Reporting of Systematic Reviews and Meta-Analysis) (31). Searches were conducted by the first author in December 2020 and updated in January 2022. The databases used were CAB Abstracts, Embase, Medline, PubMed, Scopus, Web of Science, Global Health, and the gray literature indexed in ProQuest. Search terms were adapted for the different databases.

### Search Strategy

The search strategy for CAB Abstracts, Embase, Medline, and Global health consisted of search terms and subject headings related to BVD in LMICs according to the 2020 World Bank listing of countries that are LMICs (32). We used the following search terms: “bovine viral diarrhea,” “bovine viral diarrhea virus,” “bovine viral diarrhea,” “bovine viral diarrhea virus,” “BVD,” “deveoping,” “less developed,” “under developed,” “middle income,” “low income,” “LMIC,” “LAMI,” “transitional countries,” “risk factors,” and “economic,” and these were combined with Boolean operators “OR” and “AND” (33).

### Inclusion Criteria

Inclusion criteria were defined based on the population, intervention, comparison, outcomes of an article, and study design framework (known as the PICOS framework), but in case of absence of intervention and comparison groups for PICOS, we also followed the PEO (population, exposure, outcome) framework (34). Studies analyzing exposure to BVDV in cattle from LMICs were included if they addressed questions related to risk factors for BVDV infection or if they reported health or economic outcomes for this condition. We followed the guidelines provided by the PRISMA group. We included all study types, all cattle production systems, dairy, beef, and mixed, all study designs, all years of study or publication, all studies reporting the prevalence of BVDV and its risk factors or its health and economic impacts, BVD positivity in all sample types, including blood, semen, bulk tank milk, ear-notch, skin and other tissues, LMICs using World Bank 2020 classification of countries, and studies in English or Spanish. We included articles in Spanish because of high numbers of studies identified in South America with relevant information for this review (35–39).

### Exclusion Criteria

We excluded species that were not cattle (studies specific on sheep, goats, pigs, or wild ruminants), because BVD is predominantly a disease of cattle and they are generally considered to be natural hosts (40). We excluded review articles (as they did not include any type of study, investigations or comparison groups, reportable data, and results to analyze), articles focused on cattle populations in high-income countries, and articles where the language was not English or Spanish.

### Quality Assessment of Articles

We evaluated the quality of the articles we used by risk of bias (ROB) assessment (41). Assessment of ROB helps informs readers of any potential bias in individual studies and helps facilitate their interpretation and gives an understanding of how bias might impact estimates of reliability. ROB assessment was conducted on individual studies and was based on the critical appraisal skill program (CASPS) checklist tool (42, 43). The checklist was slightly modified to ensure relevance to the review topic. All questions were imported into the Covidence version 2.0 (Veritas Health Innovation, Melbourne, Australia) software tool for systematic reviews (33). The ROB assessment consisted of 11 questions checking and assessing article quality. These included questions about the aims and design of the study, whether the target populations were well-defined, and if the results were correctly analyzed, (Supplementary Table S1). Risks were ranked based on high, low, or some concerns by at least two independent reviewers (BZS, LGG, and LHC). Low ROB meant that in the reviewer's opinion the ROB question was clearly addressed by the article. High ROB meant that the ROB question was not clearly addressed by the article or no information was provided at all, which could lead to bias. “Some concerns” meant that the there was no clear information provided to assess the ROB question in the article as assessed by the reviewers, making it difficult to judge. All articles with high risk were still eligible for data extraction and analysis (44). Although not recommended by CASPS to score articles, we needed a grading system to give an overall picture of the quality of our study articles. We graded our articles as low risk of bias (score = 2), some concerns (score = 1), and high risk of bias (score = 0), applying the criteria outlined by Yan et al. (44). All the scores were then added to give a total score for each article. The total scores ranged between 9 and 21, and a cut-off of 10 or less was agreed upon as high risk of bias article, a score between 11 and 18 was agreed upon as having some concerns, and 18 and greater was agreed upon as a low risk of bias article. Low-risk articles were then given a weighting of 2, high scoring articles were given a weighting of 0, and articles with some concerns were given a weighting of 1. All the scores and weights were then added to give each article an overall judgment using a *robvis* traffic light system as outlined by McGuinness et al. (45).

### Statistical Analysis

Statistical analyses, plotting, and mapping were conducted using the R statistic software (46, 47) in R Studio version 4.0.4 (48) using functions from the following key packages: *tidyverse* (49)*, meta* (30), and *ggplot2*, which is a part of *tidyverse*. The articles imported into Covidence were extracted as a *csv* file and imported in R Studio for cleaning and analyses. Choropleth maps were plotted using geographic information system (GIS) software and spatial libraries *sp, sf, ggmap, tmap*, and *rgdal* (50, 51). A meta-analysis was conducted using the meta packages *dmeta, metaphor*, and *meta* (30, 52). A weighted meta-analysis of animal-level prevalence of BVD reported in the reviewed articles was conducted using a logistic random-effects model (53). The random procedure incorporates an extra variance component to account for variability between the studies (heterogeneity) in addition to the within-study (sampling) variance as a result of sampling error. The weightings were calculated using the inverse variance method. The model assumes that r_ij_, which was the number of positive animals in study *i*, was a randomly distributed binomial variable where the number of animals tested is in the study was n_ij_ and the unobserved true prevalence was p_ij_ as follows: *r*_*ij*_ ~ *binom*(*n*_*ij*_, *p*_*ij*_).

The log transformation of the odds (logit) can be expressed as a linear model as follows:


$$\begin{array}{l} \log\left(\frac{p_{ij}}{1-p_{ij}}\right)=\;\beta_{0}+\;U_{0j}+C_{k} \end{array}$$

where $U_{0j}\sim N\left(0,\;\sigma_{u0}^{2}\right)$ and where β_0_ is the intercept, U_0j_ is the random effect for the intercept, and C_k_ is an addition fixed effect to account for potential heterogeneity by continent. The results of the overall meta-analysis were presented as a forest plot.

A choropleth map was plotted to show the seroprevalence of antibodies to BVDV by country. Where more than one study was available a mean of the study prevalences was used. The risk of bias descriptive results were plotted using a *robvis* traffic light plot (45) and a weighted barplot to graphically represent the ROB in each study. A sensitivity analysis was conducted by repeating the meta-analysis but removing studies with high ROB.

A descriptive analysis of reported risk factors and health impacts of BVD was conducted, and a dot matrix plot was used to display the proportions of articles reporting a given factor or an impact. Proportions of risk factor articles were calculated with confirmed risk factors/total studies.

## Results

**Figure 1:**
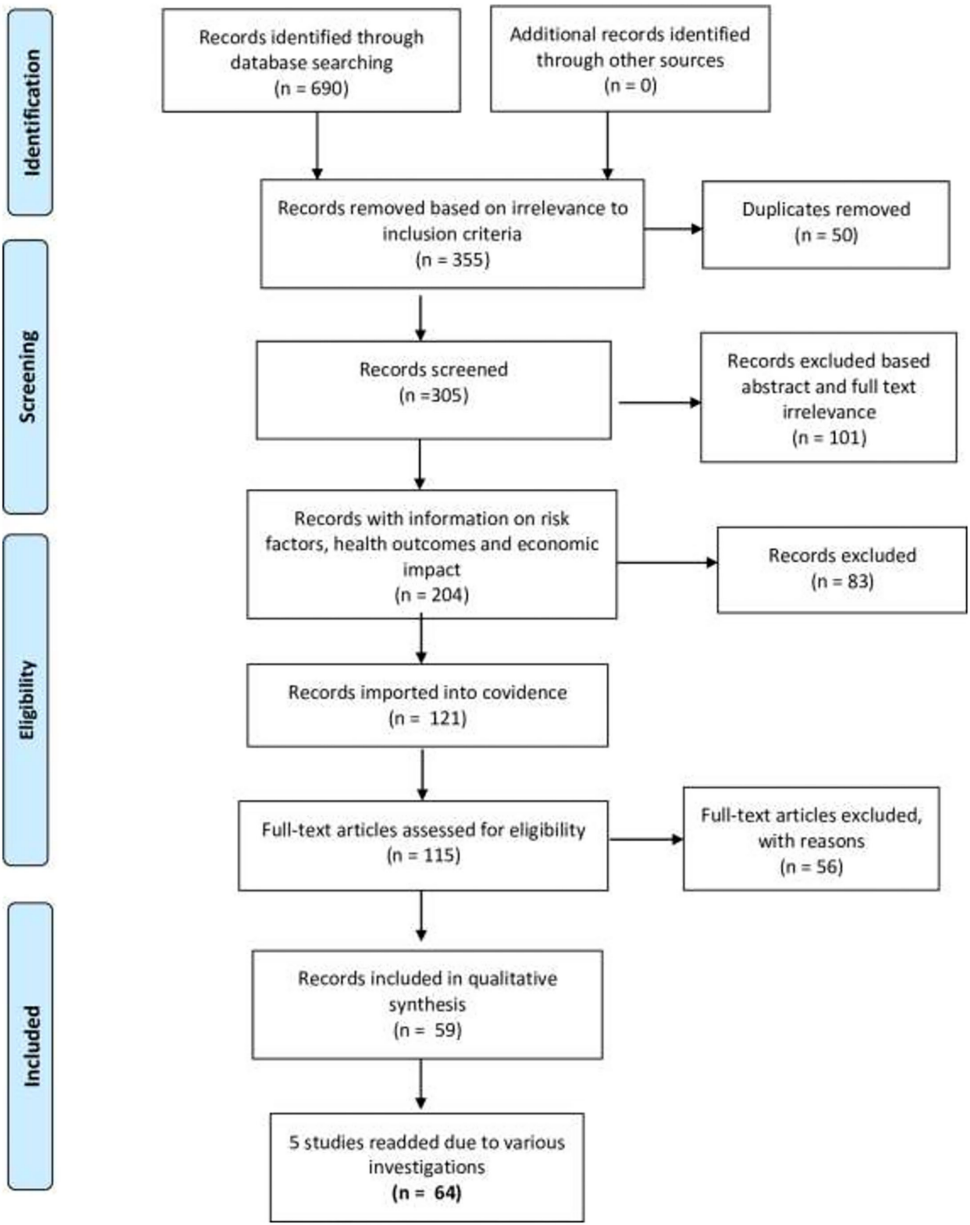
Flow diagram indicating the process of study retrieval and inclusion as described in the PRISMA statement.

There were 690 articles identified in the initial search (Figure 1). CAB Abstracts yielded 137 articles (from 1973 to 2020), Embase 28 (From 2000 to 2020), Medline 42 (from 1946 to 2020), and Global Health 26 (from 1910 to 2020). ProQuest consists of journals, theses, and dissertations from 1952 to 2020 and yielded 6,000 results. The articles were screened based on relevance to our inclusion criteria, and 335 were excluded. Out of the remaining 355, 50 duplicates were removed. The remaining 305 were further screened for title and abstract relevance and a further 101 were removed. The remaining 204 articles were screened for relevant information regarding risk factors and health or economic outcomes. A total of 121 articles were imported into Covidence. All of them passed the abstract screening stage into the full text review stage. The full text of six eligible studies was not available online, nor was it available in any holding library, and therefore these studies were eliminated. A further 56 articles were eliminated based on language other than English or Spanish, wrong outcomes, wrong settings, and for being review articles rather than primary studies. A total of 59 articles were accepted for full review, data extraction, and analysis. Data were extracted from each articles by at least two independent reviewers (BZS, LGG, and LHC) and input directly into the Covidence software tool. Consensus data were extracted for statistical analysis and plotting. Five articles out of the final 59 had various investigations, and we added the 5 separate investigations (54–58) to our 59 articles, giving us a total of 64 investigations. Therefore, it is important to note that because the five articles have included more than one prevalence type, the final sum of the number of studies is not identical to the total number of records. The process of study retrieval and selection is indicated in Figure 1.

### Characteristics of Included Studies

The final set of articles included studies from four regions, Asia, SSA, South America, and the Middle East, and covered 23 individual countries. Brazil had the highest number of studies (*n* = 12), while most countries had very few studies (Supplementary Table S2). The studies were classed as cross-sectional (*n* = 55; 84.3%), case-control (*n* = 4; 6.2%), or cohort (*n* = 4, 6.2%); in one (1.5%) case, the design was not clear from the description. The majority of the studies reported conducting random (*n* = 25), purposeful (*n* = 8), or convenience (*n* = 8) sampling, with only a few studies designing a multi-stage sampling process (*n* = 6). Some of the studies did not mention their sampling or selection process. The samples used were mainly serum, bulk tank milk, and animal tissue (ear pinnae tissue “earnotch”). BVDV antibodies or antigens were analyzed by ELISA (*n* = 50), fluorescent antibody tests (FATs) (*n* = 3), polymerase chain reactions (PCRs) (*n* = 7), serum neutralization (SN) or virus neutralization (VN) (*n* = 8) tests, or virus isolation (VI) (*n* = 1). Majority of the articles had a combination of various diagnostic tests. Vaccination was not a common practice in most of the study sites reported, and only two studies indicated that routine BVD vaccination was performed.

**Figure 2:**
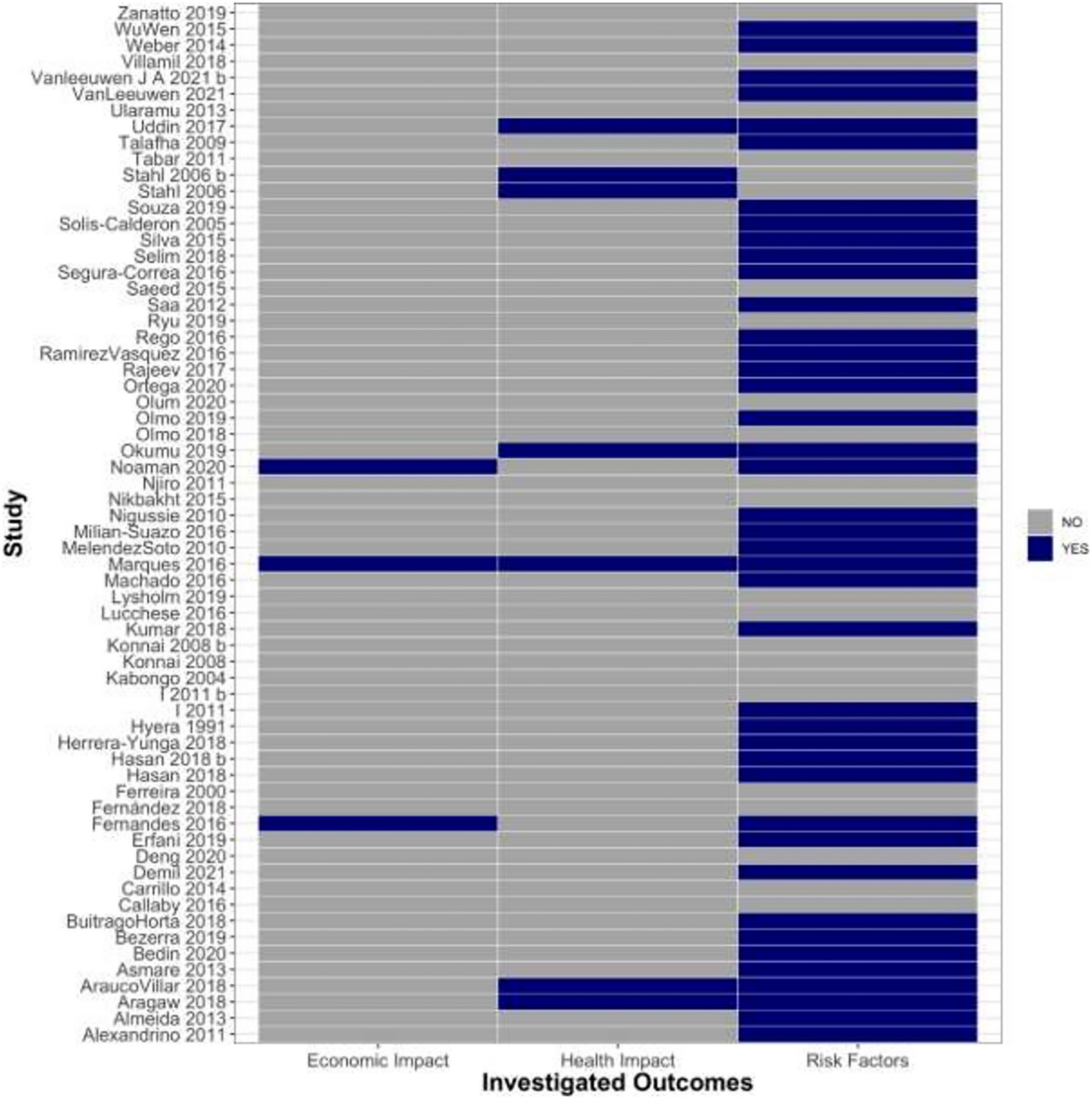
Heatmap showing breakdown of study ID and the outcomes they analyzed. The plot shows the study authors' contributions to investigation of the outcomes of interest for risk factors, health and economic impacts of BVD.

Out of the final set of studies, 41 (64.0%) reported results of analyses of risk factors, 7 (10.9%) reported results of analyses of health impact, and 3 (4.6%) studies reported analyses of economic impacts of BVD. Only 1 study (59) reported analyses of all 3 outcomes of interest (Figure 2).

### Quality Assessment

Our results show the number of studies that looked at BVD and answered the 11 questions set to address ROB (refer to list of domain questions in Supplementary Table S1). The result shows that across all studies, 50% of the studies in this review were not free of selective outcome reporting (i.e., the studies should have obtained data on all domains). More than 50% of the studies had problems with their sampling methods, study design, sampling of target population, and ethical approval. They were either not reporting correctly or not giving the authors enough information to judge.

### Prevalence: Seroprevalence

**Figure 3:**
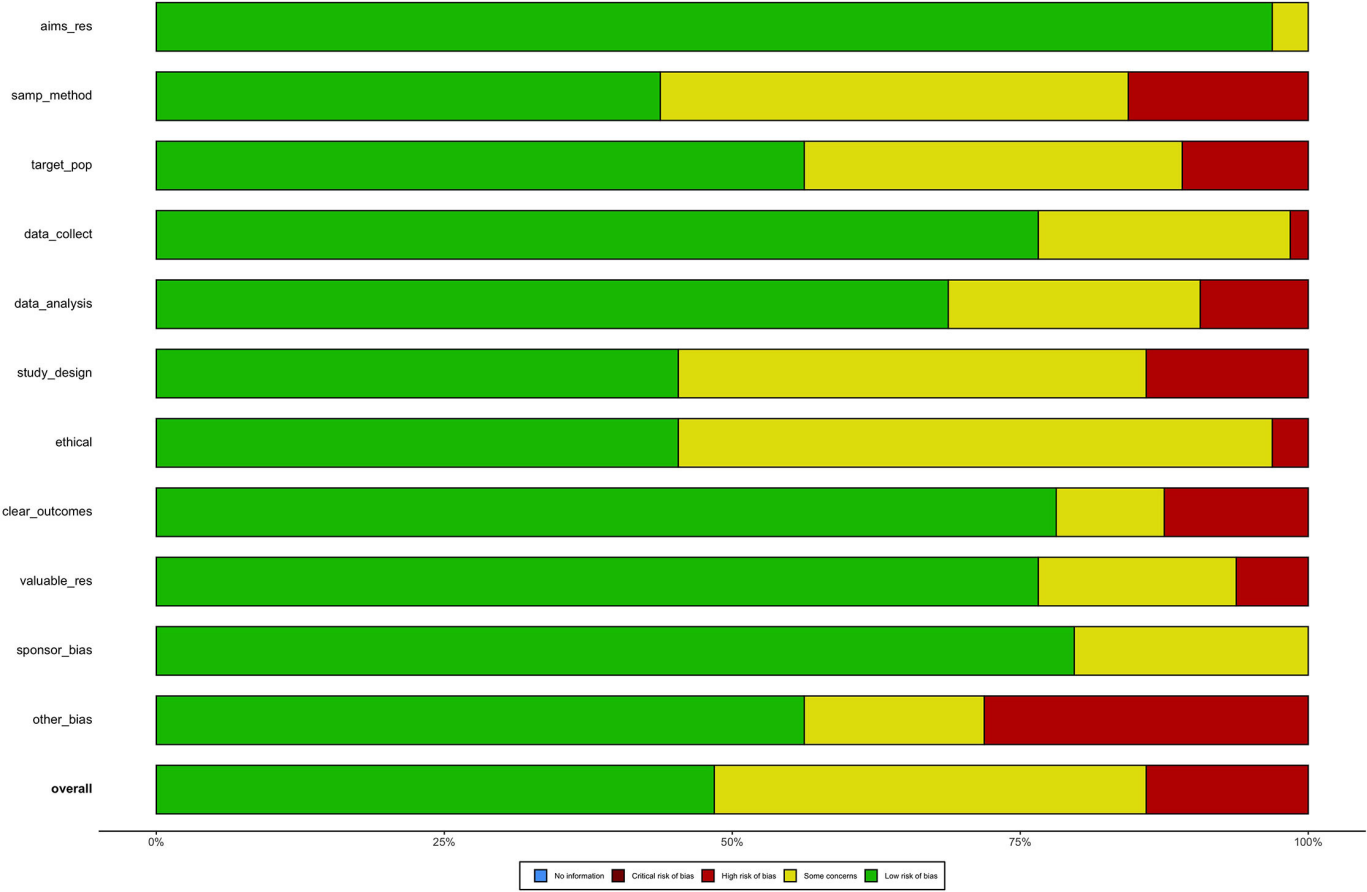
Plot showing risk of bias weights by authors judgement. The weighted plots show proportion of information with each judgement within each domain. Supplementary Table S1 explains the legend on the *y*-axis.

**Figure 4:**
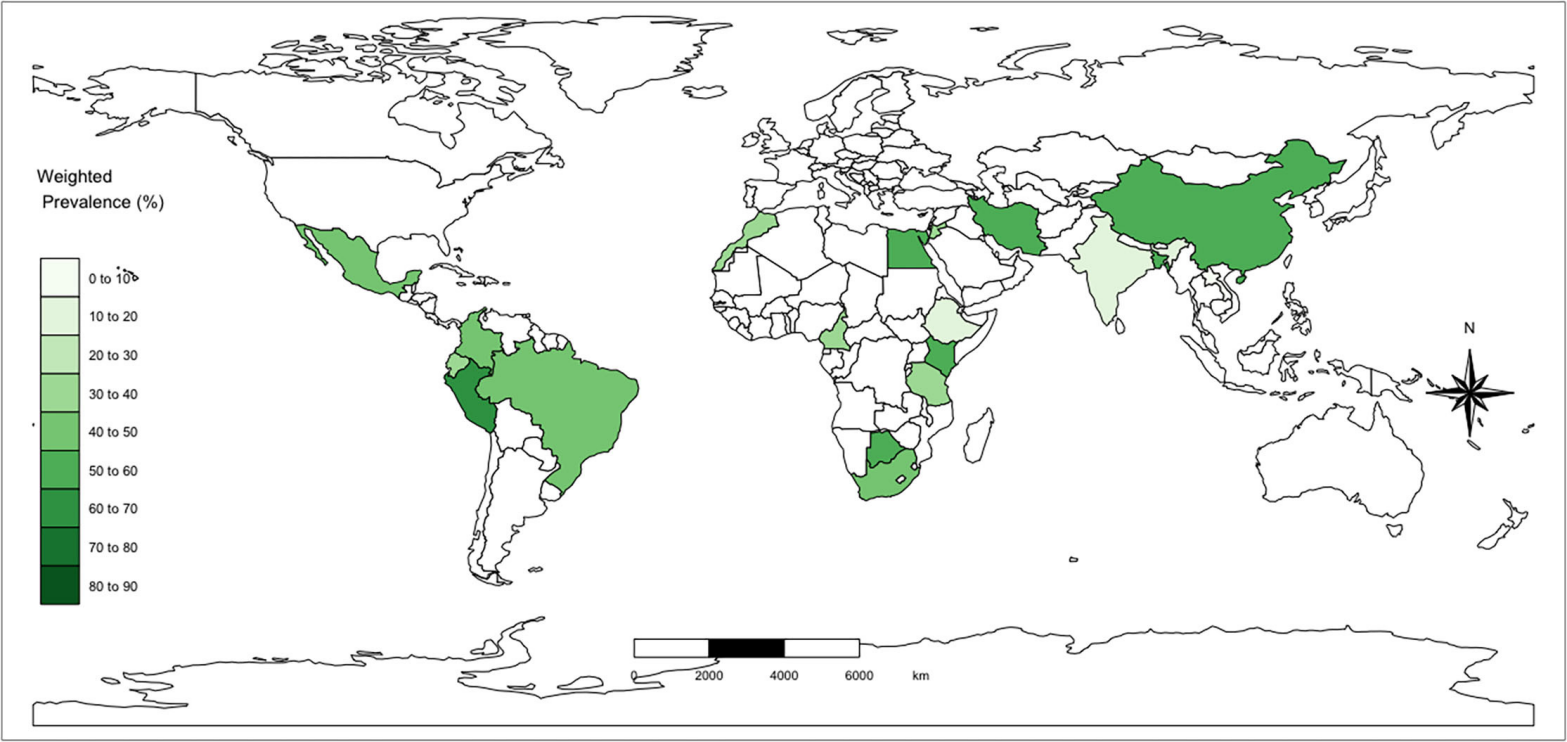
Choropleth map showing weighted mean reported seroprevalence by country. The regions included in this review were Africa, Asia, Middle East, and South/Central America.

**Figure 5:**
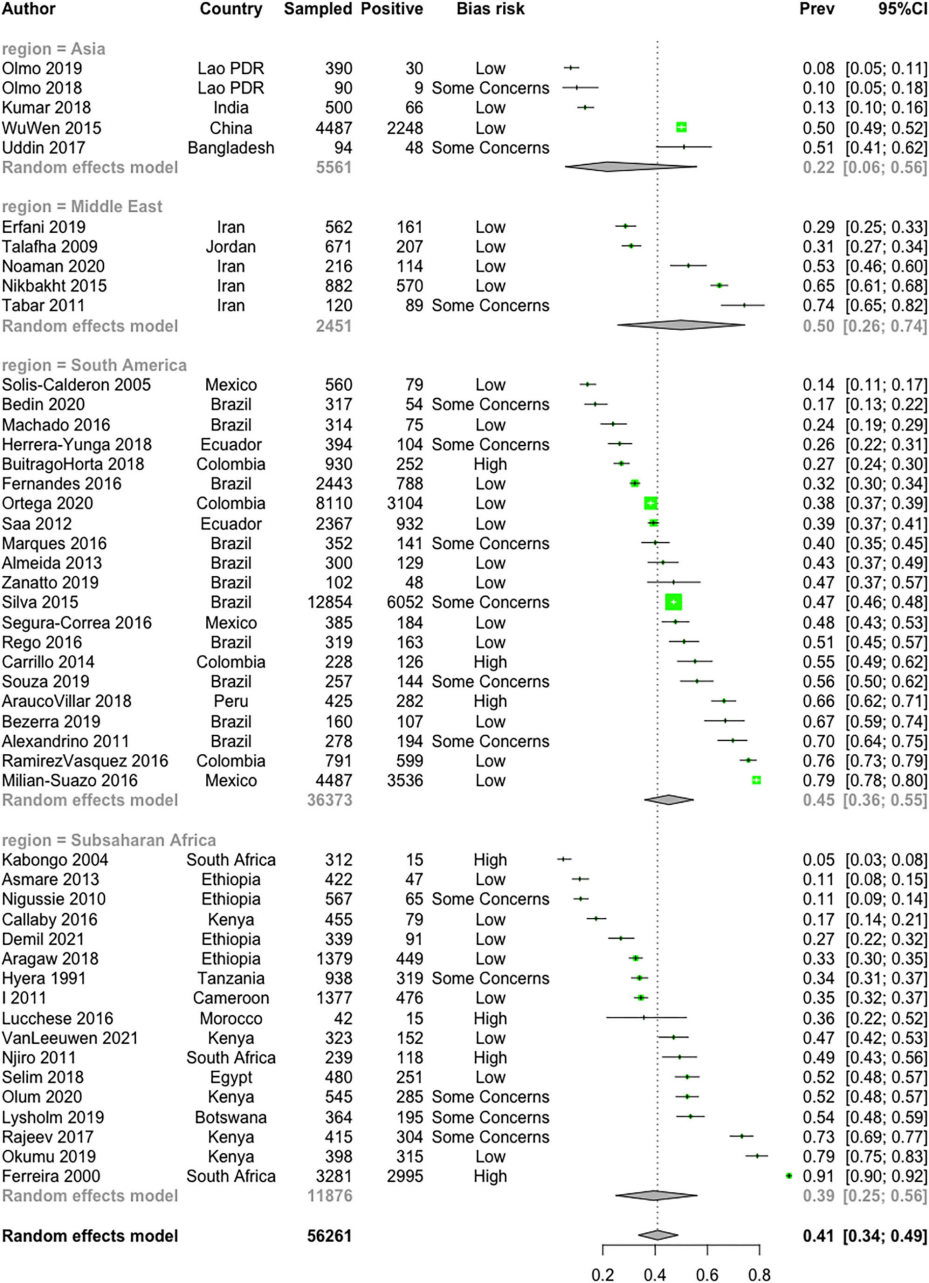
A forest plot of the weighted meta-analysis of reported BVD antibody seroprevalence by country. The plot shows the study ID, the country where the study was conducted, the sample size, the number of positive test results for antibodies to BVDV, the risk of bias rank overall, the point estimate with 95% confidence interval with the box proportional to the weighting of the fixed effect, the numerical value of the point estimate of BVD prevalence (the effect size) and the estimated 95% confidence interval (unadjusted in black, weighted adjusted in green).

All the articles gave information about prevalence. The animal-level seroprevalence was as high as 66.3% (95% CI: 61.7–70.6%) with a wide variation between regions. The country-level weighted mean seroprevalence range is between 8.2 and 66.4% and is mapped in Figure 3, while Figure 4 shows the unadjusted individual study prevalence estimates and 95% CI (based on raw numbers of positives and sample size and not accounting for any design effects or imperfect tests). Regional prevalence across Asia, South America, SSA, and the Middle East was estimated to be 21.6% (CI 6.0; 56), 45.2% (CI 35.9; 54.8), 39.4% (CI 25.5; 56.17), and 49.9% (CI 25.5; 74.3), respectively. Figure 5 presents the weighted adjusted estimates at both the continent level and overall (estimates in gray).

Weighted means are estimated by production system (Table 2) and farm type (Table 3). The majority of studies came from dairy farms with a mean of 44.5%. The mean seroprevalence estimate for other production systems was between 30 and 50% with large confidence intervals, and there was little evidence of major difference between systems (Table 2). Similarly, there appear to be little differences across farm types (Table 3). Most of the studies were conducted in small holder farms with mean seroprevalence of 41.3%.

**Table 1:** Final list of studies included in the systematic review of BVDV in LMICS ordered by year of publication.

**Study ID**	**Country of study**	**Study design**	**Sample type**	**Diagnostics**	**Test type**
VanLeeuwen, 2021	Kenya	Cross sectional study	Serum	Antibody ELISA, antigen ELISA	Antibody
Demil, 2021	Ethiopia	Cross sectional study	Serum	Antibody ELISA	Antibody
Vanleeuwen J. A., 2021b	Kenya	Cross sectional study	Serum	Antigen ELISA, antibody ELISA	Antigen
Deng, 2020	China	Cross sectional study	Ear notch	Antigen ELISA	Antigen
Noaman, 2020	Iran	Cross sectional study	Serum	Antibody ELISA	Antibody
Olum, 2020	Kenya	Cross sectional study	Serum	Antibody ELISA	Antibody
Bedin, 2020	Brazil	Cross sectional study	Serum	Antibody virus neutralization	Antibody
Ortega, 2020	Colombia	Cross sectional study	Serum	Antibody ELISA	Antibody
Zanatto, 2019	Brazil	Cross sectional study	Serum	Antibody virus neutralization	Antibody
Okumu, 2019	Kenya	Cross sectional study	Serum	Antibody ELISA	Antibody
Souza, 2019	Brazil	Cross sectional study	Serum	Seroneutralization antibody	Antibody
Erfani, 2019	Iran	Cross sectional study	Serum	Antibody ELISA	Antibody
Ryu, 2019	The Republic of Korea	Cross sectional study	Fecal	RT-PCR	Antigen
Bezerra, 2019	Brazil	Cross sectional study	Serum	Antibody ELISA	Antibody
Lysholm, 2019	Botswana	Cross sectional study	Serum	Antibody ELISA, antigen ELISA	Antibody
Olmo, 2019	Lao PDR	Cross sectional study	Serum	Antibody ELISA	Antibody
Herrera-Yunga, 2018	Ecuador	Cross sectional study	Milk	Antibody ELISA	Antibody
BuitragoHorta, 2018	Colombia	Cross sectional study	Serum	Antibody ELISA	Antibody
Olmo, 2018	Lao PDR	Cross sectional study	Serum	Antibody ELISA	Antibody
Selim, 2018	Egypt	Cross sectional study	Blood	Antibody ELISA	Antibody
Villamil, 2018	Colombia	Cross sectional study	Blood	Antigen ELISA	Antigen
Kumar, 2018	India	Cross sectional study	Serum	Antibody ELISA	Antibody
Fernández, 2018	Mexico	Cohort study	Serum	Antibody ELISA	Antibody
Aragaw, 2018	Ethiopia	Cross sectional study	Serum	Antibody ELISA	Antibody
Hasan, 2018	Iraq	Cross sectional study	Ear notch	Antigen ELISA, PCR	Antigen
AraucoVillar, 2018	Peru	Cross sectional study	Serum	Antibody ELISA	Antibody
Hasan, 2018b	Iraq	Cross sectional study	Ear notch	Antigen ELISA, PCR	Antigen
Uddin, 2017	Bangladesh	Cross sectional study	Serum	Antibody ELISA	Antibody
Rajeev, 2017	Kenya	Cross sectional study	Serum	Antibody ELISA	Antibody
Rego, 2016	Brazil	Cross sectional study	Serum	Seroneutralization antibody	Antibody
Segura-Correa, 2016	Mexico	Cross sectional study	Serum	Antibody ELISA	Antibody
Callaby, 2016	Kenya	Cohort study	Serum	Antibody ELISA	Antibody
Lucchese, 2016	Morocco	Cross sectional study	Serum	Antibody ELISA	Antibody
RamirezVasquez, 2016	Colombia	Cross sectional study	Serum	Antibody ELISA	Antibody
Milian-Suazo, 2016	Mexico	Cross sectional study	Serum	Antibody ELISA	Antibody
Marques, 2016	Brazil	Cross sectional study	Serum	Antibody ELISA	Antibody
Machado, 2016	Brazil	Case control study	Milk	Antibody ELISA	Antibody
Fernandes, 2016	Brazil	Cross sectional study	Serum	Antibody virus neutralization	Antibody
Silva, 2015	Brazil	Not specified	Serum	Antibody virus neutralization	Antibody
Saeed, 2015	Sudan	Cross sectional study	Lung	Antigen ELISA, RT-PCR, flourescent antibody test	Antigen
WuWen, 2015	China	Cross sectional study	Serum	Antibody ELISA, antigen ELISA	Antibody
Nikbakht, 2015	Iran	Cross sectional study	Serum	Antibody ELISA	Antibody
Carrillo, 2014	Colombia	Cross sectional study	Serum	Seroneutralization antibody	Antibody
Weber, 2014	Brazil	Cross sectional study	Serum	RT-PCR	Antigen
Almeida, 2013	Brazil	Cross sectional study	Milk	Antibody ELISA	Antibody
Asmare, 2013	Ethiopia	Case control study	Serum	Antibody ELISA	Antibody
Ularamu, 2013	South Africa	Cross sectional study	Tissues	rtRT-PCR, PCR	Antigen
Saa, 2012	Ecuador	Cross sectional study	Serum	Antibody ELISA	Antibody
Handel, 2011	Cameroon	Cross sectional study	Serum	Antibody ELISA, antigen ELISA	Antibody
Njiro, 2011	South Africa	Cross sectional study	Serum	Antibody ELISA	Antibody
Alexandrino, 2011	Brazil	Cross sectional study	Serum	Antibody virus neutralization	Antibody
Tabar, 2011	Iran	Case control study	Serum	Antibody ELISA, antigen ELISA	Antibody
Handel, 2011b	Cameroon	Cross sectional study	Serum	Antibody ELISA, antigen ELISA	Antigen
Nigussie, 2010	Ethiopia	Cross sectional study	Serum	Antibody ELISA	Antibody
MelendezSoto, 2010	Mexico	Case control study	Serum	Antibody ELISA	Antibody
Talafha, 2009	Jordan	Cross sectional study	Serum	Antibody ELISA	Antibody
Konnai, 2008	Philippines	Cross sectional study	Buffy coat	Antigen ELISA	Antigen
Konnai, 2008b	Philippines	Cross sectional study	Buffy coat	PCR	Antigen
Stahl, 2006	Peru	Cohort study	Milk	Antibody ELISA	Antibody
Stahl, 2006b	Peru	Cohort study	Serum	Antibody ELISA, antigen ELISA	Antigen
Solis-Calderon, 2005	Mexico	Cross sectional study	Serum	Antibody ELISA, antigen ELISA	Antibody
Kabongo, 2004	South Africa	Cross sectional study	Tissues	Antigen ELISA, immunofluorescent antibody	Antibody
Ferreira, 2000	South Africa	Cross sectional study	Serum	Indirect fluorescent antibody	Antibody
Hyera, 1991	Tanzania	Cross sectional study	Serum	Antibody virus isolation	Antibody

*It includes study ID, country of study, study design, sample type used for diagnosis, diagnostic method used to screen for bovine viral diarrhea virus exposure (antibodies) or infection (antigen), and test type (diagnostic test used in t analysis). Some of the studies performed more than one diagnostic tests.*

*ELISA, enzyme-linked immunosorbent assay. “Not specified” indicates that the authors did not specify a particular study design used in their survey. Study IDs marked with b after publication year are the 5 separate investigations added to the 59*.

**Table 2:** Summary table of weighted mean seroprevalence of antibodies to BVDV in low- and middle-income countries stratified by production system.

**Production system**	**No of papers**	**Seroprevalence**	**95%CI**
Dairy	24	44.5%	32.8–56.9
Beef	4	49.2%	11.5–87.8
Mixed	12	30.1%	23.9–56.6
Unspecified	8	33.1%	20.1–42.3

**Table 3:** Summary table of weighted mean seroprevalence of antibodies to BVDV in low-and middle income countries stratified by farming system.

**Farm type**	**No of papers**	**Seroprevalence**	**95%CI**
Small holder	24	41.3%	30.0–53.6
Semi-intensive	2	19.5%	0.0–97.2
Commercial	7	33.8%	0.9–71.0
Government	1	32.3%	30.4–34.1
Unspecified	14	47.8%	41.3–54.5

### Prevalence: Antigen Prevalence

Only nine articles reported prevalence of antigen-positive animals (i.e., infection), identifying PI or TI animals. The prevalence of antigen was 7.0% (CI 0.0; 100.0) in Asia, 10.0% (CI 0; 90.0) in SSA, 6.0% (CI 5.0; 8.0) in South America, and 9% (CI 0.0; 99.8) in the Middle East (Figure 5).

### Risk Factors

Forty-one studies (64%) reported results of risk factor analyses and the summary is presented in Figure 7. Age, breed, herd size, introducing animals, breeding practices, farm workers, and cleanliness were the risk factors studied across various production systems. Herd size was studied across all systems with proportions of 20.6%, 33.3%, and 38.4% in dairy, beef, and mixed systems, respectively. In dairy systems, age (24.1%), introducing animals (3.4%), and herd size (20.6%) were mostly studied and reported. Supplementary Table S4 presents in more detail all the risk factor groupings investigated in this review.

### Health Impact

Only seven studies (10.9%) reported on health impacts of BVDV (39, 55, 59–62). The common health impacts reported across regions and systems were abortions, and fetal complications in dairy systems. The proportion of studies that investigated abortion was 13.7%, and the proportion of studies that reported on fetal complication was 13.7% (Figure 7).

### Economic Impact

Only 3 (4.6%) studies (59, 63, 64) reported on economic losses in terms of milk yield but none quantified the losses as a cost at the herd, local, or national economic levels.

## Discussion

The objective of this review was to identify the literature reporting the prevalence of BVDV in LMICs and to summarize the risk factors associated with it, and finally its health and economic impacts. It is generally advised to limit a systematic review to a specific study design to avoid heterogeneity of study types (65). Our inclusion of a variety of study types could mean that studies are not completely comparable in the understanding of prevalence and other risk factors. Although some of our studies had small populations and sample sizes that could be a limitation in this review, they all passed the ROB analysis as low risk of bias (see Figure 3; Supplementary Figure S1 for scoring). Consequently, we have included all study types and study designs to include as much relevant information as possible, which may lead to better understanding of BVDV epidemiology in LMICs.

From the final 59 articles from which relevant data could be extracted, there were 64 studies (Table 1). Between individual studies across regions, there was an extremely wide variation in estimates of seroprevalence (Figure 5). The variation in mean seroprevalence in different farming systems could be a result of true differences in seroprevalence or due to bias estimates from poorly designed studies. As systematic random sampling was rarely conducted to take into account the existing cattle structure in different cattle populations, this could introduce some bias and give seroprevalence that is not representative of the region of study. Also, sample sizes in the different studies were not equal, as some studies had bigger populations and bigger sample sizes thereby getting a true reflection of prevalence in the region, while smaller sample size may be a limitation in obtaining the true prevalence of the disease. The findings highlight the benefit of conducting a systematic review to inform the design of future studies to obtain representative prevalence estimates from the cattle population of interest.

The variations in prevalence at the regional level highlights that there is a significant gap in information across vast parts of the global south where livestock are an important part of many household livelihoods. Based on the weighted meta-analysis using a random-effects model and given this large range in individual study seroprevalence, the regional mean seroprevalence did not vary much across South America, SSA, and the Middle East but appeared to be slightly lower in Asia (21.6%). At the country level, Peru had the highest weighted seroprevalence but with only three studies. The variation may reflect to some extent the importance and scale of cattle keeping in these areas compared to Asia and possibly the lower seroprevalence may be down to a lower cattle density in some parts of Asia. These estimates are similar to the others reported in the literature (12, 13, 66). Despite the range of seroprevalence across geographies, the overall high seroprevalence reported across LMICs suggests that there are likely inefficiencies in livestock production across these regions (4, 67). As referred to previously, many of the studies are quite limited both in numbers sampled and in terms of geographical spread, which contributes to high levels of uncertainty in all the regional weighted means. It is therefore important for future studies to fully describe their study design and population structure for estimates to be correctly interpreted.

**Figure 6:**
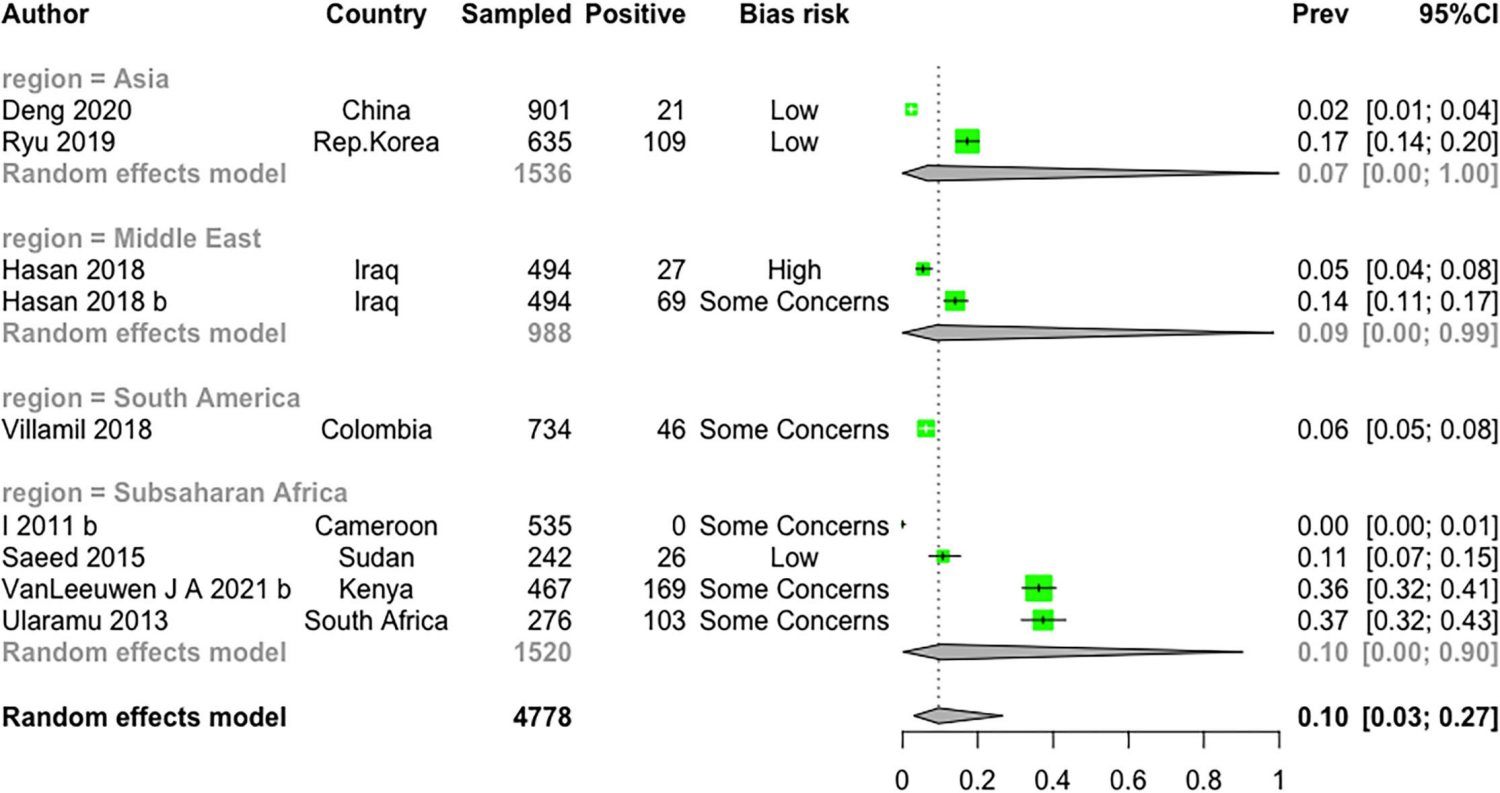
A forest plot of the weighted meta-analysis of reported BVDV antigen prevalence by country. The plot shows the study ID, the country where the study was conducted, the sample size, the number of positive test results for BVDV, the risk of bias rank overall, the point estimate with 95% confidence interval with the box proportional to the weighting of the fixed effect, the numerical value of the point estimate of BVDV prevalence (the effect size) and the estimated 95% confidence interval (unadjusted in black, weighted adjusted in green).

In addition to serological surveys, antigen studies also showed the presence of the virus in different regions, suggesting that there is ongoing transmission (Figure 6). Due to the transient nature of most BVDV infections and limited life span of PI animals, antigen-test based surveys are not necessarily useful for estimating the magnitude of transmission but can be useful for describing the molecular epidemiology of virus strains circulating (54). Few studies in this review utilized genomic tools to describe virus types present and future investigations should incorporate such testing to understand transmission networks within and between cattle populations.

**Figure 7:**
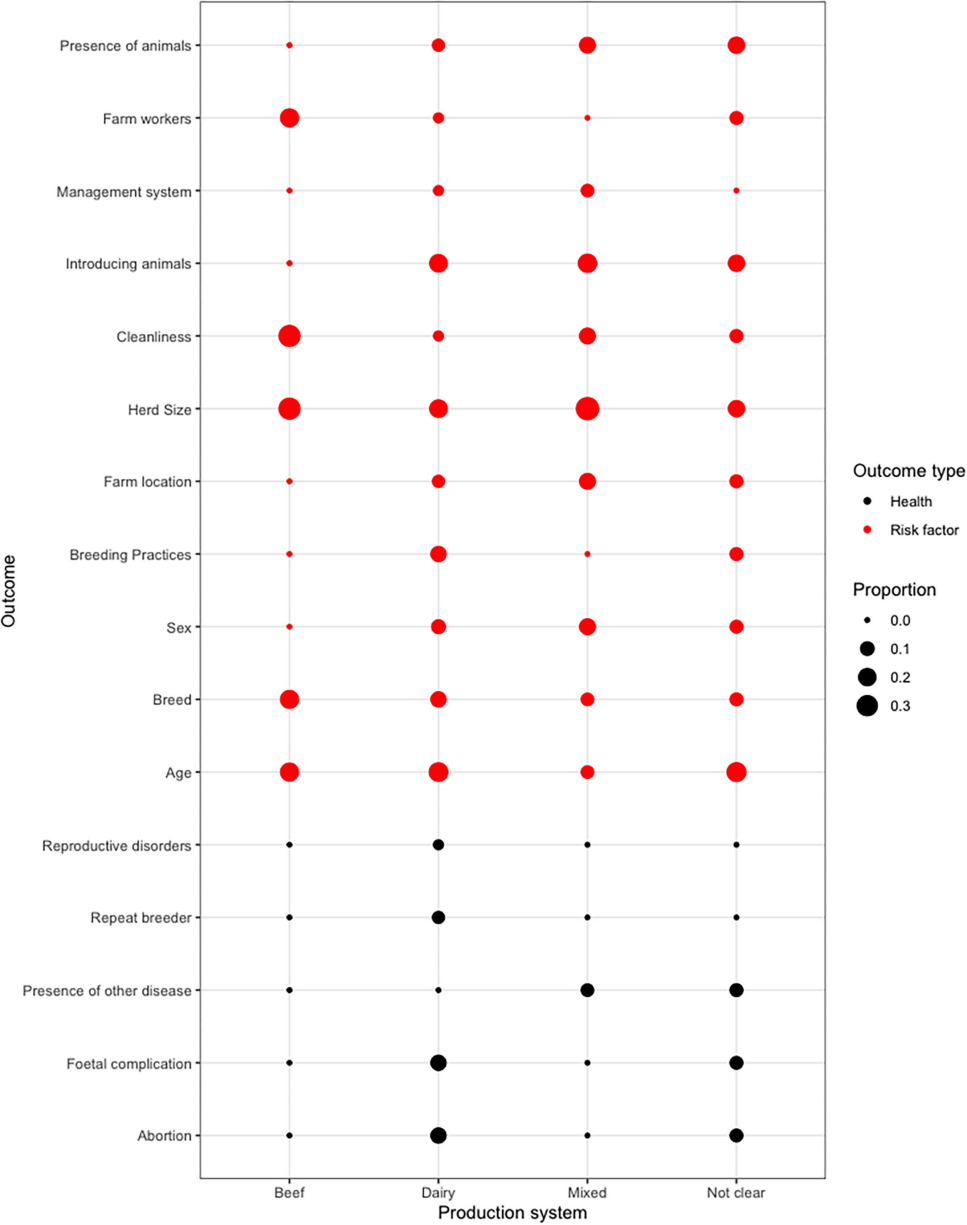
A dot matrix plot showing risk factors and health impacts reported by production system. The plot shows a meta-analysis of the pooled studies. The size of the points represents proportion of studies that reported the risk factor or health impact of BVDV for a given production system. The proportions were calculated by the formula: number of confirmed risk factor papers/total number of papers in each production system. Detailed explanations of the risk factors and how they have been grouped can be found in Supplementary Table S3.

In this review, we summarized risk factors based on 41 articles that specifically investigated risk factors. From Figure 7, we see that across production systems, herd size, introducing new animals into a farm, breeding practices, including AI, farm workers, age, and breed, were the most frequently investigated. Herd size was as a risk factor consistent in all production systems in our review, and this finding also consistent with studies conducted in high-income regions (68, 69). Most of the studies indicated that larger density stocking was a risk factor to BVD and having a smaller herd size was a protective factor. Majority of the studies that indicated herd size as a risk factor were assessed to have used a sample size that was representative in their study and a study design appropriate for their investigation. Authors discussed that herd size as a risk factor could be influenced by poor management practices, cattle density, and presence of PI animals (11, 60, 66). Larger herd size increases the risk of BVDV seropositivity (68) compared to smaller herds possibly because there is higher probability of transmission to animals in the correct stage of pregnancy ensuring generation of new PI calves that can support persistent transmission. Another reason may be as indicated by Lindberg et al. (70) that there is a low likelihood of self-clearance of virus due to continuous maintenance of the virus in herds by PIs. In contrast, smaller herds tend to have better opportunity for self-clearance and eliminate further opportunity for BVDV exposure in naïve animals.

Age was a frequently studied risk factor in beef and dairy systems. Our results show that there was a variation in directionality of age as a risk factor. Most of the studies indicated that being above 6 months increased the risk of exposure except for one study that reported that there was no association with age (71). Our review is different from a study by Houe and Meyling (72) who determined that in Danish dairy herds, incidence risk of BVDV infection was approximately similar in all age groups. The dissimilarity between our review findings and those of Houe et al. could be due to different systems of production. For example, the studies included in this review dealt mainly with smallholder and small farm sizes that do not practice vaccination or culling; therefore, animals stay in the farm until they are ready to be slaughtered. Other studies similar to ours reported that age was a significant risk factor for BVDV seropositivity with older animals being more positive to the virus (11, 66, 73). The pathogenesis of BVD shows that animals that are born as PI animals may die early because of mucosal disease or be slaughtered because of retardation usually by 2 years of age (74). However, if PI animals remain within a cattle population, they continue to maintain BVDV in the herd, exposing animals in the herd over time (73), possibly explaining the reported increasing likelihood of seropositivity with age. Because it's not clear if the risk factor is increasing or decreasing with age, further studies are needed to determine external factors which might be contributing to these varying results.

Breed was investigated as a risk factor in proportionally more studies conducted on beef systems than those conducted on dairy and mixed systems (Figure 7). It is difficult to compare between studies because there are diverse breeds in different areas. There is conflicting evidence in the literature about the importance of breed as a risk factor for example, Demil et al. (66) reported that there was no significant association between breed and BVDV seropositivity, whereas other studies have found a link between breed and BVD seropositivity. However, breed types were not consistent in the studies (62, 75). Breeds that are most susceptible have not been categorized either by farming or production systems. From our review, it was not possible to ascertain any clear pattern, so it is not possible to draw any conclusions about any specific breeds that are of higher or lower susceptibility to BVDV due to the heterogeneity of breeds in different regions. Future studies could conduct genotyping (76) to detect genomic regions associated with BVD to determine if particular breeds are more resilient or susceptible to BVDV.

Factors associated with biosecurity and hygiene were risk factors in beef and dairy systems. Farm workers, including veterinarians, milkers and other employees, and being a farm that provides milk to industries were associated with BVDV seropositivity. The high seroprevalence in dairy systems (Table 2) may also be due to the involvement of milkers, staff, and equipment during milking (73, 75, 77), which could be potential sources of contamination. Our review is similar to (75, 78, 79) and suggests that farm workers can be a potential source introducing the virus into farms. As suggested by Almeida et al. (11), farm workers can move between farms, visiting many farms in a day, and using the same clothing and instruments, and indirectly transmit the virus through plastic gloves. An important cleanliness measure to control infection from farm workers can include use of protective clothing and not allowing transportation staff to enter cow houses (69, 73). Good to medium hygiene and burying dead animals were reported as risk factors. Burning of dead animals rather than burying or disposal was reported as a protective factor, as this process successfully eliminates BVDV (80). Although not common across all production systems, some studies highlighted that breeding practices [artificial insemination (AI) and natural breeding programs] were risk factors to BVDV in dairy farms. Two of the studies reported that for AI as a risk factor, natural mating using own bulls was a protective factor (11, 81). This may indicate that infected bulls are being introduced into dairy herds for insemination, or infected semen is being distributed to AI technicians (59, 62). Introducing new animals into a herd was frequently studied and reported as a risk factor in dairy systems but not in beef systems. BVDV can be introduced into a herd by introducing new animals either through purchase or as gifts. Our result is supported by previous authors who also found that purchase of animals can introduce a PI animal into the new herds (75, 79, 82) from infected farms, thereby maintaining BVDV infection at the population level.

Exploring the motivations for herd biosecurity, cattle movements, and breeding practices would inform the design and feasibility of future control programs in LMIC settings. For example, when a study in Ireland by Graham et al. (69) found that risk of BVDV was mainly contracted from neighboring herds, movement of animals, and through farm workers a control measure was put in place to restrict cattle movement and to notify neighboring farms of their proximity to PI animals. Such measures gave farmers the opportunity to enhance their biosecurity and help in national control programs. A study conducted by Van Schaik et al. (83) suggests that understanding the risk factors and epidemiologic spread of a disease can contribute to economic benefits, which may include a more closed farming systems, rearing own young stock, and providing the cost of farm clothing.

There was little information found in studies on health implications of BVD in LMICs. The health impacts of the disease are the main cause of negative economic impacts (66). Our review found that health investigations were mainly conducted on dairy farms, with abortion and fetal complications being the more commonly reported health complications (Figure 7). However, overall few studies reported abortions or fetal complications in dairy farms, and even fewer studies reported abortions and fetal complications in beef and mixed systems. Majority of the studies that reported on abortions had their investigations on coinfections of BVDV with other diseases, so it is unclear whether BVDV was directly a cause of abortion. Previous studies reported that there are no links between BVDV and abortion (14) and have attributed the cause of abortion to be other causative agents or that cattle exposed to BVDV have cleared the infection before breeding (66). Similarly, in our review, the presence of other diseases, such as neosporosis, brucellosis, leptospirosis, and mastitis (Supplementary Table S3), was studied alongside BVDV. One of the studies by Ortega et al. (80) indicated that the presence of other diseases causing abortion were risk factors to BVDV. They reported that giving animals ivermectin, concentrated feed supplement, and organophosphates was a protective factor in farms. Although these medications would not directly treat BVD, they are possibly associated with farmers who are engaged in improved biosecurity practices. As other infections can also lead to abortion and other reproductive problems in BVD farms, it is important to consider these factors and establish a health program that includes providing prophylaxis for these infections (75). Further work will be important to investigate whether there is a relationship between abortion and other reproductive disorders and BVD in different systems as there's still not enough evidence, and these studies should aim to benchmark BVD and abortion from other causes of abortion such as return to oestrus and placental retention.

The review identified that the economic impact of BVD was minimally described and the cost implications were not quantified in production systems in LMICs. The review also highlighted the impact on milk yield in three articles, with all the studies indicating that decrease in milk yield was significantly associated with BVDV infection. The cost implications of BVD have been investigated in many high-income settings, but minimal work has been done in LMICS. Considering the importance of livestock production in livelihood incomes in LMICs, exploring whether BVD is associated with low milk production and poor production outcomes needs to be further investigated.

## Study Limitation

One of our study limitations was the inclusion of studies published only in English and Spanish. This was due to availability of speakers of the English and Spanish languages for data extraction. We excluded articles in languages not English or Spanish, as we believe these may lead to bias to studies conducted by researchers speaking other languages. This could also lead to loss of data from relevant articles that were not conducted in English or Spanish. Another limitation might be the inclusion of all study designs. It is generally advised to limit a systematic review to a specific study design to avoid heterogeneity of study types (65). We included all study types and study designs to incorporate all relevant information that may lead to better understanding of BVD epidemiology in LMICs. However, our inclusion of a variety of study types could mean that studies are not completely comparable in the understanding of prevalence and other risk factors. Some of the articles in this review had small populations and sample sizes, which could be a limitation. We believe that our ROB analysis and meta-analysis for adjusted weighing has dealt with issues of heterogeneity and comparable sample sizes.

## Conclusion

It can be concluded that BVDV is present and circulating in the regions of SSA, Asia, Middle East, and South America, and across various farming and production systems found in these regions; however, the prevalence estimates vary across these regions. The variety of risk factors of epidemiological importance is likely to be linked to various production systems within each locality. Standardized methods of estimating prevalence and the varying prevalence in diverse farming and production systems will need to be considered in trying to offer solutions for disease control strategies. The health and economic impacts associated with BVD are mostly related to reproductive performance but are poorly quantified. Future investigations should focus on quantifying the negative effect of BVD on cattle production systems in LMICs to prioritize and inform future approaches to control.

## Data Availability Statement

The original contributions presented in the study are included in the article/Supplementary Material, further inquiries can be directed to the corresponding author/s.

## Author Contributions

BZ-S, RK, and BB: writing. BZ-S, LGG, and LH-C: data extraction. LH-C, BB, and BZ-S: statistical analysis. RK, BB, EC, and BZ-S: review and editing. RK, BB, and EC: supervisors. All authors contributed to the article and approved the submitted version.

## Funding

BZ-S was supported by an EastBio PhD studentship funded by BBSRC. BB, LH-C, and EC were supported by the Bill & Melinda Gates Foundation and by a UK aid from the UK Foreign, Commonwealth, and Development Office (Grant Agreement OPP1127286) under the auspices of the Centre for Tropical Livestock Genetics and Health established jointly by the University of Edinburgh, Scotland's Rural College, and the ILRI. LGG was supported by the University of Edinburgh through the Principal's Career Development and the Edinburgh Global Scholarships.

## Conflict of Interest

The authors declare that the research was conducted in the absence of any commercial or financial relationships that could be construed as a potential conflict of interest.

## Publisher's Note

All claims expressed in this article are solely those of the authors and do not necessarily represent those of their affiliated organizations, or those of the publisher, the editors and the reviewers. Any product that may be evaluated in this article, or claim that may be made by its manufacturer, is not guaranteed or endorsed by the publisher.
