# Supplementary material for: The Epidemiology of Bovine Viral Diarrhea Virus in Low- and Middle-Income Countries: A Systematic Review and Meta-Analysis
Source: Front Vet Sci. 2022 Aug 3;9:947515. doi: 10.3389/fvets.2022.947515 (PMC9404877; doi:10.3389/fvets.2022.947515)
Supplement: Supplementary file 1 [file Data_Sheet_1.docx]

**Appendix**

**Table S1:** Summary of ROB questions and grading system

| **Study ID** | **Was there a clear statement on the aims of the research?** | **Was the recruitment or Sampling method appropriate?** | **Was the sampling frame a true or close representation of the target population?** | **Was data collected in a way that addressed the research issue?** | **Was Data Analysis Sufficiently rigorous?** | **Was the Research Design appropriate?** | **Was Ethical issues taken into consideration?** | **Is there a clear statement of finding? (Outcome reporting and completeness)** | **How Valuable is the research? (Contributions the study makes to existing knowledge)** | **Any sponsorship Funding bias?** | **Other sources of bias?** |
| --- | --- | --- | --- | --- | --- | --- | --- | --- | --- | --- | --- |
| **Vanleeuwen 2021** | Low | Low | Low | Low | Low | Low | Unsure | Low | Low | Low | Low |
| **Demil 2021** | Low | Low | Unsure | Low | Low | Low | Unsure | Low | Low | Low | Low |
| **Nigussie 2010** | Low | Low | Unsure | Unsure | Unsure | High | High | Unsure | Unsure | Unsure | Unsure |
| **Stahl 2006** | Low | Low | Low | Low | Low | Unsure | Unsure | Low | Low | Low | Low |
| **Herrera-Yunga 2018** | Low | Unsure | Unsure | Low | Unsure | Low | Unsure | Low | Low | Low | Unsure |
| **BuitragoHorta 2018** | Low | High | High | Unsure | Unsure | High | Unsure | High | Unsure | Unsure | High |
| **Rego 2016** | Low | Unsure | Low | Low | Low | Unsure | Low | Low | Low | Low | Low |
| **Deng 2020** | Low | Unsure | Low | Low | Low | Low | Low | Low | Low | Low | Unsure |
| **Carrillo 2014** | Low | High | Unsure | Unsure | High | High | Unsure | High | Unsure | Low | High |
| **Hyera 1991** | Low | Unsure | Unsure | Unsure | Unsure | Unsure | Unsure | Unsure | Low | Low | High |
| **Zanatto 2019** | Low | Unsure | Unsure | Low | Unsure | Low | Low | Low | Low | Low | Low |
| **MelendezSoto 2010** | Low | High | Unsure | Unsure | Low | Unsure | Unsure | Low | High | Low | High |
| **Okumu 2019** | Low | Low | Low | Low | Low | Low | Low | Low | Low | Low | Low |
| **Silva 2015** | Low | Unsure | Unsure | Unsure | Unsure | Low | Unsure | Low | Low | Unsure | Unsure |
| **Segura-Correa 2016** | Low | Low | Low | Low | Low | Unsure | Low | Low | Low | Low | Unsure |
| **Handel 2011** | Low | Low | Low | Low | Low | Low | Low | Unsure | Low | Low | Unsure |
| **Saeed 2015** | Low | Unsure | Unsure | Low | Low | Unsure | Low | Low | Low | Low | Low |
| **Souza 2019** | Low | High | Unsure | Unsure | Low | Low | Unsure | Low | Low | Unsure | Unsure |
| **Erfani 2019** | Low | Low | Low | Low | Low | Low | Low | Low | Low | Low | Low |
| **Ryu 2019** | Low | Unsure | Low | Low | Low | Unsure | Low | Low | Low | Low | Low |
| **Callaby 2016** | Low | Low | Unsure | Low | Low | Low | Low | Low | Low | Low | Low |
| **Kabongo 2004** | Low | High | High | Low | High | Unsure | Unsure | High | Unsure | Unsure | High |
| **Weber 2014** | Low | Unsure | Low | Low | Low | Unsure | Unsure | Low | Low | Low | High |
| **Olmo 2018** | Unsure | Unsure | Unsure | Low | Low | Unsure | Low | Low | Low | Low | High |
| **Njiro 2011** | Low | High | High | Unsure | Unsure | Unsure | Unsure | Unsure | High | Low | High |
| **Noaman 2020** | Low | Unsure | Low | Low | Low | Unsure | Low | Low | Low | Low | Low |
| **Almeida 2013** | Low | Low | Low | Low | Low | Low | Unsure | Low | Low | Low | Low |
| **Asmare 2013** | Low | Low | Low | Low | Low | Low | Unsure | Low | Low | Low | Low |
| **Lucchese 2016** | Low | Unsure | Unsure | Unsure | High | High | Unsure | Unsure | Unsure | Unsure | High |
| **Alexandrino 2011** | Low | Low | Low | Low | Low | Low | Unsure | High | Low | Low | High |
| **Selim 2018** | Low | Unsure | Low | Low | Low | Low | Unsure | Low | Low | Low | Low |
| **Villamil 2018** | Low | High | High | Low | High | High | Unsure | Low | High | Low | Low |
| **Bezerra 2019** | Low | Low | Low | Low | Low | Low | Low | Low | Low | Low | Low |
| **Uddin 2017** | Low | Unsure | Low | Low | Unsure | Unsure | Low | Low | Unsure | Low | Unsure |
| **RamirezVasquez 2016** | Low | Unsure | Low | Low | Low | Unsure | Low | Low | Low | Low | Low |
| **Milian-Suazo 2016** | Low | Low | Low | Low | Low | Low | Low | Low | Low | Low | Low |
| **Kumar 2018** | Low | Low | Low | Low | Low | Low | Low | Low | Low | Unsure | Low |
| **Fernández 2018** | Low | Low | Unsure | Unsure | High | High | Unsure | High | Unsure | Low | High |
| **Ferreira 2000** | Low | Unsure | Unsure | Unsure | Unsure | Unsure | Unsure | High | Unsure | Unsure | High |
| **WuWen 2015** | Low | Low | Low | Low | Low | Unsure | Low | Low | Low | Low | Low |
| **Olum 2020** | Low | Low | Unsure | Low | Unsure | Low | Low | Low | Low | Low | High |
| **Marques 2016** | Low | Low | Unsure | Low | Low | High | Low | Low | Low | Low | High |
| **Bedin 2020** | Low | Unsure | Low | Unsure | Unsure | Unsure | Low | High | High | Unsure | High |
| **Solis-Calderon 2005** | Low | Low | Low | Low | Low | Low | Unsure | Low | Low | Low | Low |
| **Saa 2012** | Low | Low | Low | Low | Low | Low | Low | Low | Low | Low | Low |
| **Nikbakht 2015** | Low | Unsure | Low | Low | Low | Unsure | Unsure | Low | Low | Low | Low |
| **Aragaw 2018** | Low | Low | Low | Low | Low | Low | Low | Low | Low | Low | Unsure |
| **Hasan 2018** | Low | Unsure | High | High | High | Unsure | Unsure | Low | Unsure | Unsure | High |
| **AraucoVillar 2018** | Unsure | High | High | Unsure | Unsure | High | Unsure | High | Unsure | Unsure | High |
| **Lysholm 2019** | Low | Unsure | Low | Low | Low | High | Low | Low | Low | Low | High |
| **Ortega 2020** | Low | Low | Low | Unsure | Low | Low | High | Low | Low | Low | Low |
| **Rajeev 2017** | Low | High | Unsure | Low | Low | Low | Unsure | Low | Low | Low | Low |
| **Machado 2016** | Low | Unsure | Low | Low | Low | Low | Low | Low | Low | Low | Low |
| **Talafha 2009** | Low | Low | Low | Low | Low | Low | Low | Low | Low | Low | Low |
| **Olmo 2019** | Low | Low | Low | Low | Low | Unsure | Low | Low | Low | Low | Low |
| **Konnai 2008** | Low | High | Unsure | Low | Unsure | Unsure | Unsure | Low | Low | Low | Low |
| **Fernandes 2016** | Low | Unsure | Low | Low | Low | Low | Low | Low | Low | Low | Low |
| **Tabar 2011** | Low | Unsure | Low | Low | Low | Unsure | Unsure | Low | Unsure | Low | Unsure |
| **Ularamu 2013** | Low | Unsure | High | Low | Low | Unsure | Unsure | Low | Low | Unsure | Low |
| **Stahl 2006 b** | Low | Low | Low | Low | Low | Unsure | Unsure | Low | Low | Low | Low |
| **Handel 2011 b** | Low | Low | Low | Low | Low | Low | Low | Low | Low | Low | Low |
| **Vanleeuwen J A 2021 b** | Low | Low | Low | Low | Low | Low | Unsure | Low | Low | Low | Low |
| **Hasan 2018 b** | Low | Unsure | Unsure | Low | Low | Unsure | Unsure | Low | Low | Unsure | Low |
| **Konnai 2008 b** | Low | Unsure | Unsure | Low | Unsure | Unsure | Unsure | Unsure | Low | Low | Low |

**Table S2:** Table showing countries in which the study was conducted, and number of investigations carried out in each country.

**Table S3:** A list of risk factors as identified by our studies grouped under themes of risk factors with similar characteristics.

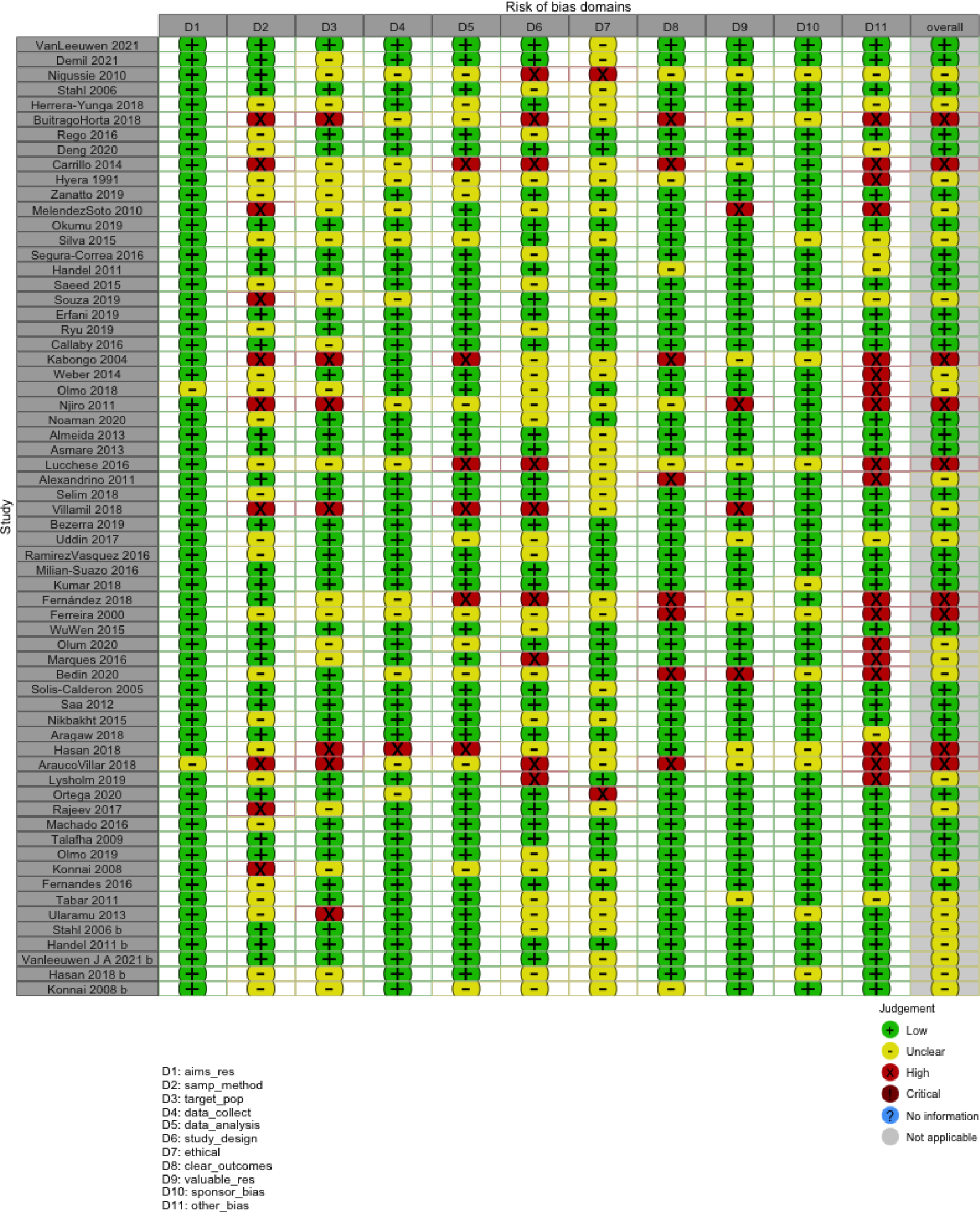


**Figure S1:** Plot showing risk of bias overall summary by authors judgement. The plot tabulates the judgement for each study in each domain by a traffic light system.
